# Knowledge of transgender and gender-diverse healthcare among resident physicians: A study in a northeastern Brazilian tertiary hospital

**DOI:** 10.20945/2359-4292-2025-0013

**Published:** 2025-09-28

**Authors:** Vivianne Almeida da Nóbrega, Erik Trovão Diniz, Norma Arteiro Filgueira

**Affiliations:** 1 Divisão de Endocrinologia e Metabologia, Hospital das Clínicas, Universidade Federal de Pernambuco, Recife, PE, Brasil; 2 Divisão de Clínica Médica, Hospital das Clínicas, Universidade Federal de Pernambuco, Recife, PE, Brasil

**Keywords:** Transgender people, medical education, health equity

## Abstract

**Objective:**

Transgender and gender-diverse (TGD) refers to people whose gender identity does not correspond to the sex assigned to them at birth. This study evaluated the knowledge of medical residents at a tertiary hospital in northeastern Brazil regarding healthcare for the TGD population.

**Materials and methods:**

This cross-sectional, single-center observational study surveyed medical residents at a tertiary hospital in northeastern Brazil in 2023. It utilized a self-developed online questionnaire, which residents completed voluntarily and anonymously. Descriptive statistics, chi-square analyses, and multivariate logistic regression were applied to the data.

**Results:**

A total of 107 residents completed the questionnaire (40.83% of the eligible cohort); most were clinicians (69.15%). All participants identified as cisgender. Nearly all participants considered it important to understand healthcare for TGD patients. About half reported prior education on the topic; gynecology, obstetrics, and endocrinology residents (specialists) demonstrated the highest rates (p = 0.0009). Approximately 40% of the participants were unaware of where to refer TGD people for specialized care in hormone therapy and gender-affirming surgeries (p = 0.007). Lack of experience (p = 0.002) was the primary reason among the 30 residents who felt insecure about providing healthcare to TGD patients.

**Conclusion:**

Residents acknowledge the importance of this field in their practice but demonstrate a lack of specific knowledge and prior education.

## INTRODUCTION

Gender identity is defined as each individual’s recognition of their own gender. The term “transgender and gender-diverse” (TGD) refers to people whose gender identity does not correspond to the sex assigned to them at birth (^1^,^2^). For some, their gender identity does not fit within the well-known male/female binary. “Transexual” is an older term that denotes individuals who seek or have completed medical interventions to facilitate gender transition. Such interventions modify physical and social characteristics to align with gender identity (^1^).

Transgender people constitute approximately 2% of Brazil’s adult population, equivalent to nearly 3 million people (^3^). They face transphobia, stigmatization, and physical and psychological violence. In 2022, Brazil’s National Council of Justice data indicated a 35.2% increase in violence against the LGBTQIA+ community (^4^). Furthermore, the estimated average life expectancy of Brazilian TGD people is approximately 35 years (^5^). Between October 2023 and September 2024, 106 TGD deaths were documented in Brazil, almost one-third of the global total (^6^). Although under-reporting is widespread, the country’s high figures may reflect systematic monitoring by national organizations (^6^).

Brazil’s healthcare system addressed these disparities by adopting the National Policy for Comprehensive Health of Lesbians, Gays, Bisexuals, Transvestites, and Transgender in 2011. This policy seeks, among other objectives, to expand equitable access to the *Sistema Único de Saúde* (SUS [Unified Health System]) and to meet the health needs of this population (^7^).

The SUS “Transsexualization Process”, established in 2008 and updated in 2013, specifies within the scope of Specialized Care the structural and staffing requirements for outpatient and inpatient services (^8^). Outpatient services encompass clinical follow-up and hormone therapy delivered by, at minimum, a psychiatrist or psychologist, a social worker, an endocrinologist or general practitioner, and a nurse. Inpatient services provide gender-affirming surgeries and require a multidisciplinary team comprising a urologist, a gynecologist or plastic surgeon, a nurse, a psychiatrist or psychologist, an endocrinologist, and a social worker.

Brazil’s Federal Council of Medicine Resolution nº 2.265/2019 addresses healthcare for TDG patients, emphasizing the importance of comprehensive care for this population (^9^). It states that hormone therapy can be administered from the age of 16, while gender reassignment surgery is allowed from the age of 18.

The TGD people still face limited understanding from healthcare providers and occasional refusal of care, contributing to disparities compared to cisgender people (^2^). They experience poorer physical health, including a higher prevalence of obesity, diabetes mellitus, HIV, and mental health issues such as depression and suicide (^2^,^10^-^12^). Undergraduate and residency curricula frequently overlook TGD healthcare, hindering doctors from building and enhancing their knowledge on the subject and perpetuating service deficiencies (^13^). These curricular gaps affect multiple specialties and encompass sexual health prevention (*e.g.*, sexually transmitted infections and cancer screening), mental health management, and appropriate terminology (^10^). Such shortcomings mirror the negative experiences reported by TGD people, particularly in underdeveloped countries (^14^,^15^).

Few Brazilian studies have investigated residents’ knowledge of TGD healthcare. To date, no research has evaluated resident physicians at Hospital das Clínicas of the Federal University of Pernambuco, despite its status as one of the few SUS-accredited transsexualization centers in the country offering both multidisciplinary outpatient and surgical care (^16^). Therefore, this study assessed residents’ knowledge of TGD healthcare at this tertiary university hospital, compared findings across specialties, and estimated the proportions of those who had received relevant training and who felt confident managing TGD patients.

## MATERIALS AND METHODS

This cross-sectional, single-center observational study surveyed medical residents at a tertiary hospital in Recife, Pernambuco (northeastern Brazil), from July to December 2023. Convenience sampling included physicians enrolled in medical residency programs during this period; those who declined participation were excluded.

Based on Brazil’s Federal Council of Medicine Resolution nº 2.265/2019 and adapted from related studies, we employed a self-authored, non-validated questionnaire (^2^,^17^). It comprised closed-ended items addressing sociodemographic characteristics and healthcare knowledge for TDG patients (**Appendix A**).

Participants received an electronic invitation via a messaging application, individually and within groups, to complete the questionnaire. Responses were provided voluntarily and anonymously through Microsoft Forms (Microsoft, USA) after electronic informed consent was obtained. Questionnaire data were entered into Microsoft Excel (v. 2020, Microsoft, USA), and statistical analyses were conducted with R software (R project, New Zealand). Analyses comprised descriptive statistics of means and proportions. Group differences were assessed with the chi-square test. Associations between variables were evaluated by multivariate logistic regression, generating odds ratios (ORs) with 95% confidence intervals. Statistical significance was set at *p* < 0.05. Participants were stratified into three specialty groups: (a) clinicians of all clinical specialties, except for endocrinology, psychiatry, intensive care medicine, and pediatrics and its subspecialties, except pediatric endocrinology; (b) non-clinicians of all surgical specialties, plus radiology and pathology; (c) specialists in gynecology and obstetrics (OB/GYN), urology, and endocrinology that routinely manage TGD patients and comprise the referral team for outpatient and inpatient care in the SUS’ Transsexualization Process.

The study complied with Brazil’s Federal Council of Medicine Resolution nº 466/2012 (^14^), and was approved by the Research Ethics Committee (CAAE nº 70669623.2.0000.8807). All study procedures were initiated only after ethics approval and completion of informed consent.

## RESULTS

A total of 107 residents completed the questionnaire (40.83% of the eligible cohort), 54.8% of the hospital’s clinicians, 32.5% of specialists, and 23% of non-clinicians (**Table 1**). All participants identified as cisgender. Nearly all participants considered knowledge of TGD healthcare important in terms of beliefs and attitudes. Almost every resident expressed concern about using each patient’s chosen name and pronouns (**Table 2**). Nearly all respondents recognized the right of transgender patients to be addressed by their social name in SUS facilities, and approximately 80% understood the concept of non-binary gender identity. About 60% were aware of where to refer TGD patients for hormone therapy or gender reassignment surgery.

**Table 1:** Sociodemographic characteristics of surveyed medical residents

Characteristics	n (%)
Average age (y)	28.23
Sex	
Cisgender woman	63 (58.87)
Cisgender man	44 (41.13)
Specialties	
Clinics	74 (69.15)
Non-clinical	20 (18.69)
Gynecology and obstetrics, endocrinology	13 (6.54)

**Table 2:** Beliefs, attitudes, and self-reported knowledge of medical residents about care for transgender and gender-diverse patients

Item assessed	Frequency of responses (n, %)	
	Yes	No
When providing care, do you ensure that you use pronouns consistent with the patient’s gender identity?	106 (99.06)	1 (0.94)*
When providing care, do you ensure that you use the patient’s social name?	105 (98.13)	2 (1.87)**
Were you aware that transgender patients have the right to be addressed by their social name in SUS services?	104 (97.20)	3 (2.80)
Do you understand the concept of a non-binary gender identity?	87 (81.30)	20 (18.7)
Do you consider it important to understand healthcare for TGD patients?	104 (97.20)	3 (2.80)
Have you received previous education on healthcare for TGD patients?	55 (51.40)	52 (48.6)
Do you know where to refer TGD patients for hormone therapy or gender reassignment surgery?	65 (60.75)	42 (39.25)
(OB/GYN, endocrinology, urology, or family and community medicine only) Have you ever received guidance on prescribing gender-affirming hormone therapy?	11 (84.61)	2 (15.39)
(Surgical specialties or OB/GYN only) Do you feel able to give a brief description of surgical options for gender-affirming procedures in transgender men?	4 (18.18)	18 (81.82)
(Surgical specialties or OB/GYN only) Do you feel able to give a brief description of surgical options for gender-affirming procedures in transgender women?	6 (27.27)	16 (72.73)

TGD: transgender and gender-diverse; OB/GYN: gynecology and obstetrics.

* For ideological and/or religious reasons.

** They did not believe it was important for good service.

**Figure 1:**
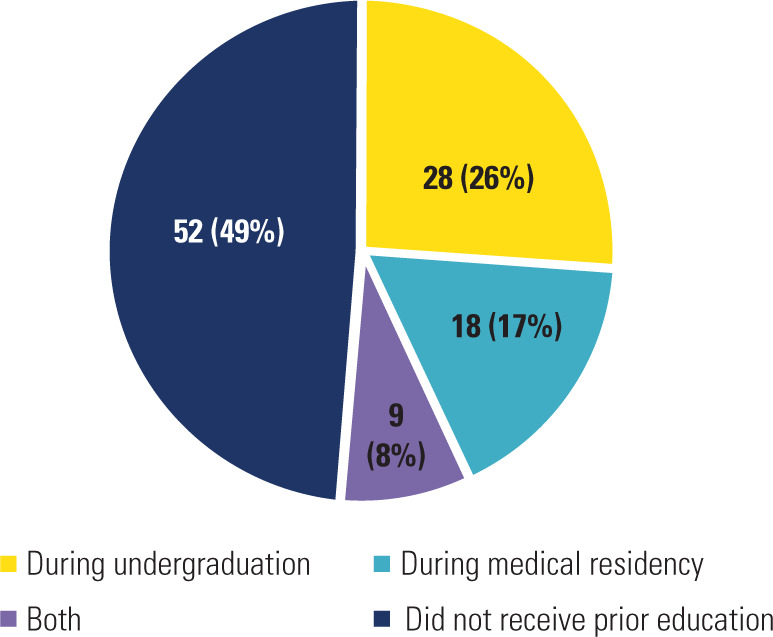
When participants first learned about healthcare for transgender and gender-diverse patients.

Although approximately half of the residents had received prior instruction on TGD health, predominantly during undergraduate training (**Figure 1**), most respondents lacked adequate knowledge of the ethical-legal framework governing transgender care (**Table 3**). Only roughly 20% correctly identified breast cancer screening recommendations for transgender men post-mastectomy, although around 30% of specialists responded accurately. Conversely, most participants correctly cited cervical cancer screening guidelines for transgender men with a cervix. Knowledge of mental health parameters was also generally appropriate.

**Table 3:** Knowledge of medical residents about care for transgender and gender-diverse patients

Item assessed	Correct answer (n, %)
A male patient outwardly presents himself as a woman (through clothes, hairstyle, and behavior). Does this mean that this person identifies as a transgender woman? (N*)	62 (57.94%)
A transgender man is an individual assigned female at birth who identifies as a man. (Y*)	105 (98.13%)
Is transgender identity classified as a psychiatric disorder? (N*)	103 (96.26%)
What is the life expectancy of the TGD people in Brazil? (Lower than the general population*)	103 (96.26%)
Are there regulations in Brazil that govern medical procedures for TGD people? (Y*)	87 (81.30%)
According to Brazil’s Federal Council of Medicine Resolution No. 2.265/2019, at what minimum age may TGD people begin hormone therapy in Brazil? (16 years old*)	42 (39.25%)
According to the same resolution, at what minimum age is gender reassignment surgery permitted in Brazil? (18 years old*)	48 (44.86%)
Are TGD people at increased risk of psychiatric disorders such as depression and anxiety? (Y*)	107 (100%)
Are TGD people at increased risk of suicidal ideation? (Y*)	105 (98.13%)
After undergoing mastectomy, should transgender men continue routine breast cancer screening if they remain within the indicated age range? (N*)	22 (20.56%)
Should transgender men who retain a cervix continue routine cervical cancer screening according to Brazilian guidelines and age criteria? (Y*)	96 (89.71%)
Is concomitant use of antiretroviral therapy a contraindication to hormone therapy for body modification in TGD patients? (N*)	65 (60.74%)

TGD: transgender and gender-diverse; Y: yes; N: no. *Correct answer.

Fewer than 50% correctly identified the minimum age to start hormone therapy and perform gender reassignment surgery in Brazil. Specialists reported the highest frequency of prior instruction (*p* = 0.0009) and were more likely to know the appropriate age to start hormone therapy (*p* = 0.009) and the referral pathways for gender-affirming interventions (*p* = 0.007; **Table 4**). The OB/GYN and endocrinology residents most frequently answered correctly that antiretroviral therapy is not a contraindication to gender-affirming hormone therapy (92.3%; *p* = 0.036).

**Table 4:** Beliefs, attitudes, and self-reported knowledge of medical residents about care for transgender and gender-diverse patients, stratified by specialty

Item assessed	Frequency of affirmative responses (n, %)			
	Clinicians (n = 74)	Non-clinicians (n = 20)	Specialists* (n = 13)	p
When providing care, do you ensure that you use pronouns consistent with the patient’s gender identity?	100%	95%	100%	0.111
When providing care, do you ensure that you use the patient’s social name?	97.29%	100%	100%	0.634
Were you aware that transgender patients have the right to be addressed by their social name in SUS services?	95.94%	100%	100%	0.502
Do you understand the concept of a non-binary gender identity?	79.72%	85%	84.61%	0.821
Do you consider it important to understand healthcare for TGD patients?	98.64%	90%	100%	0.093
Have you received previous education on healthcare for TGD patients?	44.59%	45%	100%	0.0009
A male patient outwardly presents himself as a woman (through clothes, hairstyle, and behavior). Does this mean that this person identifies as a transgender woman?	35.13%	30%	46.15%	0.488
A transgender man is an individual assigned female at birth who identifies as a man.	97.29%	100%	100%	0.634
Is transgender identity classified as a psychiatric disorder?	2.7%	10%	0%	0.234
Are there regulations in Brazil that govern medical procedures for TGD people?	78.37%	85%	92.3%	0.835
Do you know where to refer a TGD patient for hormone therapy or gender reassignment surgery?	54.05%	60%	100%	0.007
Are TGD people at increased risk of psychiatric disorders such as depression and anxiety?	100%	100%	100%	-
Are TGD people at increased risk of suicidal ideation?	97.29%	100%	100%	0.634
After undergoing mastectomy, should transgender men continue routine breast cancer screening if they remain within the indicated age range?	50%	60%	61.53%	0.247
Should transgender men who retain a cervix continue routine cervical cancer screening according to Brazilian guidelines and age criteria?	86.4%	95%	100%	0.558
Is concomitant use of antiretroviral therapy a contraindication to hormone therapy for body modification in TGD patients?	0%	0%	0%	0.036
What is the current life expectancy of the transgender population in Brazil?	94.59%	100%	100%	0.762
According to Brazil’s Federal Council of Medicine Resolution No. 2.265/2019, at what minimum age may TGD people begin hormone therapy in Brazil?	36.48%	30%	69.23%	0.0009
According to the same resolution, at what minimum age is gender reassignment surgery permitted in Brazil?	40.54%	45%	69.23%	0.171

TGD: transgender and gender-diverse. *Gynecology, obstetrics, and endocrinology.

**Figure 2:**
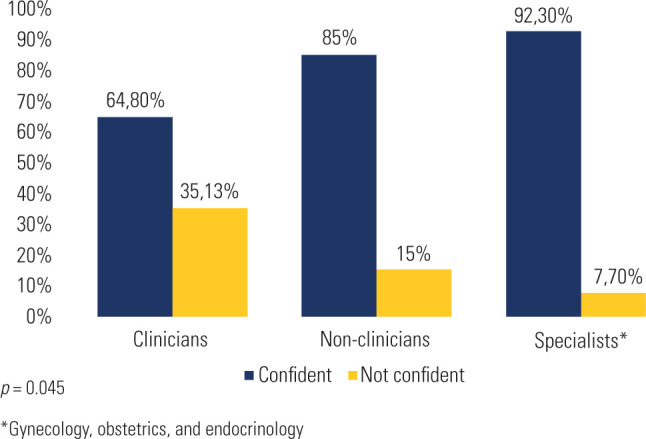
Confidence in providing care to transgender and gender-diverse patients among surveyed residents.

Approximately 70% of the residents were confident in caring for TGD patients, notably higher among specialists (92.3%; *p* = 0.045; **Figure 2**). Compared with non-specialists, specialists demonstrated a non-significant trend toward greater confidence (OR 5.35, 95% CI 0.66-43.13, *p* = 0.115). Prior education significantly increased confidence (OR 3.46, 95% CI 1.40-8.55, *p* = 0.007). After multivariate logistic regression, previous education remained an independent predictor (OR 2.88, 95% CI 1.11-7.43, *p* = 0.029), whereas specialist status no longer reached significance (OR 2.82, 95% CI 0.32-24.99, *p* = 0.351). Lack of experience (*p =* 0.002) was the primary reason among the 30 residents who felt insecure about providing healthcare to TGD patients, followed by insufficient prior knowledge; no respondent cited personal, moral, or religious objections.

## DISCUSSION

This study is among the first to evaluate Brazilian medical residents’ knowledge of healthcare for TGD individuals. Despite the hospital’s status as a national referral center for this population, most participants reported insufficient prior training and demonstrated inadequate knowledge. Clinicians yielded a higher response rate than non-clinicians. The lower participation of surgeons may reflect a limited interest in the topic and could have introduced selection bias. All participants identified as cisgender, and most self-reported as cisgender women, corroborating the 2023 Brazilian resident demographic in which approximately 56% are female (^18^). Brazilian data on the number of TGD residents remain unavailable.

Nearly all respondents considered competence in TGD healthcare important. A North American survey in both clinical and surgical specialties consistently reported that 60.6% consider it important to understand the subject for their current practice and 74.2% for future practice (^19^). In Internal Medicine, there is little data on healthcare provision to TGD people. Nevertheless, most residents recognize the topic’s relevance for their practice (^13^), a finding consistent with this study. Despite evident interest and willingness to learn more about it, they receive insufficient training in caring for TGD people, leading to persistent knowledge gaps, resulting in knowledge gaps (^10^,^13^).

Brazil’s National Curriculum Guidelines, which guide the structuring and developing of undergraduate medical education, mention “gender diversity” twice: once as a dimension of healthcare and once as a consideration when performing physical examination techniques ethically and respectfully (^20^). In contrast, Brazil’s National Commission for Medical Residency’s competency matrices, documents that define residency curricula, omit TGD healthcare. Among the specialties comprising SUS’ Transsexualization Process teams, only psychiatry mentions gender identity, with a pathologizing approach to the issue. It requires second-year residents to “master the diagnosis and treatment […] of disorders related to gender identity” (^21^-^23^).

In this context, the importance of incorporating topics related to healthcare for TGD patients into medical residency programs is emphasized. To this end, it is suggested that: 1) such topics be formally included in the curricula of the specialties that comprise the reference team in the SUS Transsexualization Process, with encouragement for continuing education; 2) spaces for discussion on the care of the TGD population be included, whether through clinical meetings, lectures, or case discussions, allowing for the sharing of experiences and integration between the medical and multiprofessional teams; 3) health promotion actions be carried out in hospitals, disseminating information on the healthcare of TGD patients to medical specialties that are not part of the reference team for this type of care; 4) academic research on the various issues related to the health of the TGD population be encouraged, aiming to reduce existing knowledge gaps in the scientific field.

In this regard, it is recommended that each reference center in the SUS Transsexualization Process conduct studies to assess healthcare professionals’ knowledge about caring for the TGD population. This would enable more targeted and specific changes, based on the deficiencies identified in each location. Furthermore, it is proposed that regional and national events be organized to provide opportunities for knowledge sharing among different centers, including their challenges and strengths. The goal is to develop strategies to minimize potential shortcomings in TGD patient care, which could be brought to the attention of the Ministries of Health and Education to support broader improvements in public health.

In identifying barriers to healthcare access for TGD people in Minas Gerais (southeastern Brazil) using semi-structured interviews with these individuals, professional training was highlighted as very relevant. The interviewees perceived gaps in technical qualifications and the welcoming nature of the system (^24^).

Although the clinical importance of TGD health is well recognized, approximately half of the residents reported previous formal instruction, a proportion consistent with published evidence. Correspondingly, a Pakistani study conducted at a leading university found that 51% of medical students had received formal education on TGD health (^14^). In North America, roughly 39% of physicians across multiple specialties reported comparable training (^25^). Specialty residents most frequently reported previous instruction obtained during either undergraduate education or residency. Surveying OB/GYN residents showed that 49.4% had received formal TGD health education, a significant training barrier (^26^).

Nevertheless, most respondents were confident in caring for TGD patients, particularly OB/GYN and endocrinology residents. A similar pattern (80%) was observed in a tertiary-care center in a rural setting in the USA (^27^). Conversely, only 37.9% reported confidence in TGD care at institutions affiliated with Michigan State University, which used an online questionnaire aimed at residents and preceptors (^19^). The participants justified it by a lack of professional experience and prior knowledge. A North American study also implicated inadequate exposure and formal training as causes for deficits in the healthcare of TGD people (^19^). The findings confirm that prior education positively correlates with residents’ confidence, underscoring the need for structured training opportunities.

Knowledge gaps persisted in specialized content: a small proportion of specialist doctors answered correctly about the cessation of breast cancer screening in transgender men undergoing mastectomy as a body modification surgery. Fewer than 20% of surgical residents felt capable of counseling transgender men on gender reassignment surgery options, and about 30% felt prepared to advise transgender women. Moreover, fewer than one-third of clinicians, surgeons, residents, and attending doctors demonstrated competence in the subject (^19^). Another study of predominantly North American OB/GYN residents reported an intermediate level of comfort and competence, including counseling on gender reassignment surgery (^28^)

Regarding mental health, residents correctly recognized that TGD people have higher risks of suicidal ideation and developing depression and anxiety. This finding corroborates earlier reports in which 92% of respondents noted an elevated risk of suicidal ideation, and 97.5% acknowledged increased risks of depression and suicide (^13^,^19^). Although approximately 40% of participants did not know where to refer TGD patients for hormone therapy or gender reassignment surgery, they worked in a tertiary referral center. Parallel gaps were observed in 2021, with 34.7% of clinicians aware of referral pathways for hormone therapy and 39% for surgery (^19^).

In conclusion, this is the first study that assesses Brazilian residents’ competence in TGD health at a tertiary referral hospital, demonstrating that they value the topic yet lack specialized knowledge of ethical-legal issues and cancer screening.

Despite our promising findings, this study has several limitations. First, the sample was small, particularly among non-clinicians; no urologists completed the questionnaire, a specialty that theoretically represents one of the most capable of providing specialized care. Second, the over-representation of clinicians may have introduced response bias, which may have overestimated the perceived knowledge and confidence. Third, the absence of standardized and validated questionnaires for Brazil limits external validity and the unavailability of similar national studies on the subject.

Hence, more space for discussion and training on healthcare in medical residency must be created to foster equitable, comprehensive care and to reduce stigma and violence against TGD populations. In addition, the Brazilian Ministry of Education should incorporate TGD competencies into residency matrices, and further research is required to understand better and characterize training deficits and propose strategies to remedy these gaps.

## Data Availability

datasets related to this article will be available upon request to the corresponding author.

## APPENDIX A – Instrument Used for Data Collection

**Gender:**

- Cisgender man
- Transgender man
- Cisgender woman
- Transgender woman
- Non-binary

**Age:**

**Medical residency program:**

- Clinical specialties:
  ○ Internal Medicine
  ○ Endocrinology
  ○ Infectious Diseases
  ○ Family and Community Medicine
  ○ Others: which one?
- Surgical specialties:
  ○ General Surgery
  ○ Urology
  ○ Others: which one?
- Gynecology and Obstetrics
- Pediatrics
- Psychiatry
- Anesthesiology

**Questions:**

1. When providing care, do you ensure that you use pronouns consistent with the patient’s gender identity?
  - Yes
  - No
  - If you answered “no”, what is the reason?
    - For ideological and/or religious reasons
    - Because I don’t consider it important for good care
    - Other reasons: which?_________
    - Prefer not to answer
2. When providing care, do you ensure that you use the patient’s social name?
  - Yes
  - No
  - If you answered “no”, what is the reason?
    - For ideological and/or religious reasons
    - Because I don’t consider it important for good care
    - Other reasons: which?_________
    - Prefer not to answer
3. Were you aware that transgender patients have the right to be addressed by their social name in SUS services?
  - No
  - Yes
4. Do you understand the concept of a non-binary gender identity?
  - Yes
  - No
5. Do you consider it important to understand healthcare for TGD patients?
  - Yes
  - No
6. Have you received previous education on healthcare for TGD patients?
  - Yes
  - If yes, when?
    - During undergraduation
    - During medical residency
    - Both
  - No
7. How confident do you feel in providing healthcare to transgender and gender-diverse patients?
  - Very confident
  - Confident
  - Moderately confiante
  - Slightly confidente
  - Not at all confidente
8. If you do not feel confident in providing healthcare to transgender and gender-diverse patients, please indicate the main reason why.
  - Lack of prior knowledge on the subject
  - Lack of professional experience with this population
  - Personal, moral, or religious reasons
  - Prefer not to answer
9. A male patient outwardly presents himself as a woman (through clothes, hairstyle, and behavior). Does this mean that this person identifies as a transgender woman?
  - Yes
  - No
  - I don’t know
10. A transgender man is an individual assigned female at birth who identifies as a man.
  - True
  - False
11. Is transgender identity classified as a psychiatric disorder?
  - Yes
  - No
12. What is the life expectancy of the TGD people in Brazil?
  - Same as that of the general population
  - Lower than the general population
  - I don’t know
13. Are there regulations in Brazil that govern medical procedures for TGD people?
  - Yes
  - No
14. According to Brazil’s Federal Council of Medicine Resolution No. 2.265/2019, at what minimum age may TGD people begin hormone therapy in Brazil?
  - 16 years
  - 18 years
  - 21 years
  - I don’t know
15. According to the same resolution, at what minimum age is gender reassignment surgery permitted in Brazil?
  - 18 years
  - 21 years
  - I don’t know
16. Do you know where to refer a TGD patient for hormone therapy or gender reassignment surgery?
  - Yes
  - No
17. Are TGD people at increased risk of psychiatric disorders such as depression and anxiety?
  - Yes
  - No
  - I don’t know
18. Are TGD people at increased risk of suicidal ideation?
  - Yes
  - No
  - I don’t know
19. If you are from the fields of Endocrinology, Gynecology and Obstetrics or Urology: have you ever received any guidance on how to prescribe hormone replacement therapy for transgender patients?
  - Yes
  - No
  - I am not from these fields
20. If you are from the surgical specialties or Gynecology and Obstetrics: do you feel capable of succinctly explaining to your patient the options for gender-affirming surgery for trans men?
  - Yes
  - No
  - I am not from these fields
21. If you are from the surgical specialties or Gynecology and Obstetrics: do you feel capable of succinctly explaining to your patient the options for gender-affirming surgery for trans women?
  - Yes
  - No
  - I am not from these fields
22. After undergoing mastectomy, should transgender men continue routine breast cancer screening if they remain within the indicated age range?
  - Yes
  - No
  - I don’t know
23. Should transgender men who retain a cervix continue routine cervical cancer screening according to Brazilian guidelines and age criteria?
  - Yes
  - No
  - I don’t know
24. Is concomitant use of antiretroviral therapy a contraindication to hormone therapy for body modification in TGD patients?
  - Yes
  - No
  - I don’t know

## References

[1] Hembree WC, Cohen-Kettenis PT, Gooren L, Hannema SE, Meyer WJ, Murad MH (2017). Endocrine treatment of gender-dysphoric/gender-incongruent
persons: An Endocrine Society clinical practice guideline. J Clin Endocrinol Metab..

[2] Coleman E, Radix AE, Bouman WP, Brown GR, de Vries ALC, Deutsch MB (2022). Standards of Care for the Health of Transgender and Gender
Diverse People, Version 8. Int J Transgender Health.

[3] Spizzirri G, Eufrásio R, Lima MCP, de Carvalho Nunes HR, Kreukels BPC, Steensma TD (2021). Proportion of people identified as transgender and non-binary
gender in Brazil. Sci Rep..

[4] Conselho Nacional de Justiça, Programa das Nações Unidas para o
Desenvolvimento (2022). Discriminação e violência contra a
população LGBTQIA+ : relatório da pesquisa
[Internet].

[5] Benevides BG (2025). Dossiê: assassinatos e violências contra travestis e
transexuais brasileiras em 2024. ANTRA (Associação Nacional de
Travestis e Transexuais) [Internet].

[6] TMM numbers – TvT [Internet].

[7] Brazil (2011). Portaria nº 2.836, de 1º de dezembro de 2011. Institui, no âmbito
do Sistema Único de Saúde (SUS), a Política Nacional de
Saúde Integral de Lésbicas, Gays, Bissexuais, Travestis e
Transexuais (Política Nacional de Saúde Integral
LGBT).

[8] Brazil (2013). Portaria nº 2.803, de 19 de novembro de 2013. Redefine e amplia o
Processo Transexualizador no Sistema Único de Saúde
(SUS).

[9] Brazil (2020). Resolução CFM nº 2.265, de 9 de janeiro de 2020.
Dispõe sobre o cuidado específico à pessoa com
incongruência de gênero ou transgênero e revoga a
Resolução CFM nº 1.955/2010.

[10] Streed CG, Hedian HF, Bertram A, Sisson SD (2019). Assessment of Internal Medicine Resident Preparedness to Care for
Lesbian, Gay, Bisexual, Transgender, and Queer/Questioning
Patients. J Gen Intern Med..

[11] Dragon CN, Guerino P, Ewald E, Laffan AM (2017). Transgender Medicare Beneficiaries and Chronic Conditions:
Exploring Fee-for-Service Claims Data. LGBT Health.

[12] De Brier N, Van Schuylenbergh J, Van Remoortel H, Van den Bossche D, Fieuws S, Molenberghs G (2022). Prevalence and associated risk factors of HIV infections in a
representative transgender and non-binary population in Flanders and
Brussels (Belgium): Protocol for a community-based, cross-sectional study
using time-location sampling. PLoS One.

[13] Johnston CD, Shearer LS (2017). Internal Medicine Resident Attitudes, Prior Education, Comfort,
and Knowledge Regarding Delivering Comprehensive Primary Care to Transgender
Patients. Transgender Health.

[14] Lee JL, Huffman M, Rattray NA, Carnahan JL, Fortenberry JD, Fogel JM (2022). “I Don’t Want to Spend the Rest of my Life Only Going to a Gender
Wellness Clinic”: Healthcare Experiences of Patients of a Comprehensive
Transgender Clinic. J Gen Intern Med..

[15] Martins RS, Saleh R, Kamal H, Gillani M, Merchant AAH, Munir MM (2020). The need for transgender healthcare medical education in a
developing country. Adv Med Educ Pract..

[16] Indicadores – Cnes [Internet].

[17] Stroumsa D, Shires DA, Richardson CR, Jaffee KD, Woodford MR (2019). Transphobia rather than education predicts provider knowledge of
transgender health care. Med Educ..

[18] Scheffer M, Guilloux AGA, Miotto BA, Almeida CJ, Guerra A, Cassenote A (2023). Demografia Médica no Brasil 2023 [Internet].

[19] Kelly-Schuette K, Little A, Davis AT, Mensah FK, Wright GP (2021). Transgender Surgery: Perspectives across Levels of Training in
Medical and Surgical Specialties. Transgender Health.

[20] Brazil (2014). Ministério da Educação.

[21] Brazil (2022). Ministério da Educação. Matrizes de
Competências. Programa de residência médica em
Psiquiatria.

[22] Brazil (2019). Ministério da Educação. Aprova a matriz de
competências dos Programas de Residência Médica em
Urologia.

[23] Brazil (2019). Ministério da Educação. Aprova a matriz de
competências dos Programas de Residência Médica em
Endocrinologia.

[24] Araujo S (2024). Explorando as complexidades e os desafios do acesso à
saúde para pessoas trans em Minas Gerais: um estudo qualitativo
após uma década da implementação do processo
transexualizador no Sistema Único de Saúde. Revista do Sistema Único de Saúde do Brasil.

[25] Kent D, Perry K, Vanier C, Havins B (2022). Assessing Comfort of Physicians to Provide Transgender-Specific
Care. Transgender Health.

[26] Burgart JM, Walters RW, Shanahan M (2022). Transgender Education Experiences among Obstetrics and Gynecology
Residents: A National Survey. Transgender Health.

[27] Rowan SP, Lilly CL, Shapiro RE, Kidd KM, Elmo RM, Altobello RA (2019). Knowledge and Attitudes of Healthcare Providers Toward
Transgender Patients within a Rural Tertiary Care Center. Transgender Health.

[28] Qin LA, Estevez SL, Radcliffe E, Shan WW, Rabin JM, Rosenthal DW (2021). Are Obstetrics and Gynecology Residents Equipped to Care for
Transgender and Gender Nonconforming Patients? A National Survey
Study. Transgender Health.

